# End-Stage Renal Disease Patients Lose a Substantial Amount of Amino Acids during Hemodialysis

**DOI:** 10.1093/jn/nxaa010

**Published:** 2020-01-31

**Authors:** Floris K Hendriks, Joey S J Smeets, Natascha J H Broers, Janneau M X van Kranenburg, Frank M van der Sande, Jeroen P Kooman, Luc J C van Loon

**Affiliations:** 1 Department of Human Biology, NUTRIM School of Nutrition and Translational Research in Metabolism, Maastricht University Medical Centre+, Maastricht, The Netherlands; 2 Department of Internal Medicine, NUTRIM School of Nutrition and Translational Research in Metabolism, Maastricht University Medical Centre+, Maastricht, The Netherlands; 3 Division of Nephrology, Department of Internal Medicine, Maastricht University Medical Centre+, Maastricht, The Netherlands

**Keywords:** protein, muscle wasting, nutrient loss, kidney disease, chronic hemodialysis patients

## Abstract

**Background:**

Poor nutritional status is frequently observed in end-stage renal disease patients and associated with adverse clinical outcomes and increased mortality. Loss of amino acids (AAs) during hemodialysis (HD) may contribute to protein malnutrition in these patients.

**Objective:**

We aimed to assess the extent of AA loss during HD in end-stage renal disease patients consuming their habitual diet.

**Methods:**

Ten anuric chronic HD patients (mean ± SD age: 67.9 ± 19.3 y, BMI: 23.2 ± 3.5 kg/m^2^), undergoing HD 3 times per week, were selected to participate in this study. Spent dialysate was collected continuously and plasma samples were obtained directly before and after a single HD session in each participant. AA profiles in spent dialysate and in pre-HD and post-HD plasma were measured through ultra-performance liquid chromatography to determine AA concentrations and, as such, net loss of AAs. In addition, dietary intake before and throughout HD was assessed using a 24-h food recall questionnaire during HD. Paired-sample *t* tests were conducted to compare pre-HD and post-HD plasma AA concentrations.

**Results:**

During an HD session, 11.95 ± 0.69 g AAs were lost via the dialysate, of which 8.26 ± 0.46 g were nonessential AAs, 3.69 ± 0.31 g were essential AAs, and 1.64 ± 0.17 g were branched-chain AAs. As a consequence, plasma total and essential AA concentrations declined significantly from 2.88 ± 0.15 and 0.80 ± 0.05 mmol/L to 2.27 ± 0.11 and 0.66 ± 0.05 mmol/L, respectively (*P* < 0.05). AA profiles of pre-HD plasma and spent dialysate were similar. Moreover, AA concentrations in pre-HD plasma and spent dialysate were strongly correlated (Spearman's ρ *=* 0.92, *P* < 0.001).

**Conclusions:**

During a single HD session, ∼12 g AAs are lost into the dialysate, causing a significant decline in plasma AA concentrations. AA loss during HD can contribute substantially to protein malnutrition in end-stage renal disease patients. This study was registered at the Netherlands Trial Registry (NTR7101).

## Introduction

Patients with end-stage renal disease fail to adequately remove metabolic waste products and excess fluids from the body (1). To prevent lethal consequences of waste product accumulation, hemodialysis (HD) is employed to replace 10–15% of renal clearance capacity (2). However, patients undergoing chronic hemodialysis (CHD) treatment typically develop impairments in physical function due to a decline in lean tissue mass, cardiorespiratory capacity, and muscle strength (3–5). Though muscle and strength loss can be part of the normal aging process, the progressive loss of skeletal muscle mass is remarkably accelerated in CHD patients (6, 7). Skeletal muscle wasting in CHD patients can be attributed to various factors, including inflammation, malnutrition, and nutrient loss during each HD session (8–10).

Amino acids (AAs) are among the nutrients lost in the dialysate during HD and are of key importance for muscle maintenance (10, 11). Previous work from our laboratory (12–15). as well as that from many others (16–21), has shown that skeletal muscle protein turnover is highly responsive to postprandial increases in plasma AA concentrations. In both healthy and clinical populations the postprandial rise in plasma AA concentrations stimulates muscle protein synthesis rates and inhibits protein breakdown, allowing net muscle protein accretion (14, 22). In CHD patients, muscle protein synthesis as well as breakdown rates are stimulated during HD (23, 24). Previous studies have shown that loss of AAs during HD causes a decline in plasma AA concentrations in fasted patients (11, 25–29). Moreover, HD induces a negative net forearm AA balance in fasting patients, which may be indicative of muscle proteolysis (24).

In contrast to clinical practice in North America, most CHD patients in Europe are allowed to eat and drink during their HD treatment (30). There is an ongoing debate on the implementation of dietary protein intake during HD to counterbalance the HD-induced decline in plasma AA concentrations in routine care, as some nephrologists cite concerns regarding patient safety and increased staff burden. Moreover, it remains to be established whether habitual food intake before and during HD increases the subsequent loss of AAs in the dialysate. Previous estimates may, therefore, not accurately reflect AA loss in CHD patients consuming their habitual diet during HD.

Therefore, we selected 10 CHD patients to participate in a study in which we obtained blood samples and spent dialysate during HD to assess the selective AA loss in the dialysate. Plasma and dialysate AA concentrations were measured to calculate individual AA extraction rates and to evaluate the relationship between basal plasma AA concentrations, food intake, and AA extraction during HD. This study provides insights into the AA extraction and nutritional requirements of CHD patients consuming their habitual diet during HD.

## Methods

### Subjects

Ten patients with a urine production below 100 mL/d, undergoing HD 3 times per week with high-flux membranes for at least 6 mo, were recruited through the outpatient population visiting the HD department of Maastricht University Medical Centre+, Maastricht, The Netherlands. Patients with an active infection, cognitive disorder, or missed HD session in the last month prior to the study period were excluded. Patients’ characteristics are presented in **Table 1**. Patients were informed of the nature of the experimental procedures prior to providing written informed consent. The current study was approved by the Medical Ethical Committee of the Maastricht University Medical Centre+ and registered at the Netherlands Trial Registry (NTR7101). The applied study design complies with the standards outlined in the most recent version of the Helsinki Declaration.

**TABLE 1:** Characteristics of included CHD patients^1^

	Patients
Age, y	67.9 ± 19.3
Gender, *n*	
Male	7
Female	3
Cause of ESRD, *n*	
Hypertension	4
Diabetes mellitus	2
Autoimmune	2
Other	2
Dialysis vintage, mo	46.8 ± 28.4
Height, cm	166 ± 9
Weight, kg	64.4 ± 15.9
BMI, kg/m^2^	23.2 ± 3.5
Lean tissue index, kg/m^2^	11.7 ± 1.7
Handgrip strength, kg	24.5 ± 11.7
Serum albumin, g/L	33.5 ± 2.6

1 All values are expressed as mean ± SD, *n* = 10. CHD, chronic hemodialysis; ESRD, end-stage renal disease.

### Study design

A single test day per patient was scheduled during the patients’ second or third weekly HD session. Before HD, patients’ handgrip strength and body composition were measured using a mechanical dynamometer (Jamar) and a body composition monitor (Fresenius Medical Care), as described before (31). Directly before and after a 4-h HD session, blood was sampled from the arterial side of the arteriovenous shunt for analysis of plasma AA concentrations. Throughout HD, spent dialysate was continuously collected at a rate of 1.00 L/h in a container using a reversed injection pump (Alaris GW). Every 30 min the collection container was replaced with a new one. After homogenization of the contents of the filled container, a sample of the collected dialysate was obtained.

### Hemodialysis treatment

Patients’ prescribed blood (300–400mL/min) and dialysate flow rates (500–600 mL/min) were used during HD. The desired ultrafiltration volume (mean 1.75 ± 0.71 L) was determined by the treating nephrologist. HD sessions were performed with high-flux polysulfone (*n* = 7; FX-100, Fresenius Medical Care) and polynephron (*n* = 3; ELISO-17H, Nipro Medical Corporation) membranes, with surface areas of 2.2 and 1.7 m^2^, respectively. Dialysate composition used was equal for all HD sessions and contained sodium (138 mM), potassium (2.00 mM), calcium (1.50 mM), magnesium (0.50 mM), chloride (109 mM), bicarbonate (32.0 mM), and glucose (1.00 g/L).

### Food intake

Patients were encouraged to consume their habitual diet before and during the test day. Habitual food intake during HD consisted mainly of homemade sandwiches, cookies, coffee, and tea. During the 4th h of the HD session, dietary intake records of the participants were acquired through a 24-h food recall questionnaire. One researcher, who had received training by a licensed dietician, carefully instructed patients on how to perform the food recall questionnaire. All ingested foods and beverages were reported in household measurements or specified as portion sizes. Subsequently, energy and macronutrient intakes were calculated using free available software from the Dutch Nutrition Centre (mijn.voedingscentrum.nl) based upon product specifications provided by food suppliers and the Dutch Food Consumption Database 2016 (NEVO; RIVM) (32). Reported food intake was calculated for the HD session and the 24-h period.

### Plasma AA concentrations

Blood samples were collected in EDTA-containing tubes and centrifuged at 3500 × *g* at 4°C for 10 min to obtain plasma. Aliquots of plasma were frozen in liquid nitrogen and stored in a freezer at −80°C until further analysis. For determination of plasma AA concentrations, 50 µL of blood plasma was deproteinized using 100 µL of 10% 5-sulfosalicylic acid (SSA) with 50 µM of the metabolomics AA mix MSK-A2 internal standard (Cambridge Isotope Laboratories). Subsequently, 50 µL of ultra-pure demineralized water was added and this solution was centrifuged at 14,000 × *g* at 4°C for 15 min. After centrifugation, 10 µL of the supernatant was added to 70 µL borate reaction buffer (Waters). In addition, 20 µL of AccQ-Tag derivatizing reagent solution (Waters) was added, after which the mixture was heated to 55°C for 10 min. AA profiles in the derivative were determined by ultra-performance liquid chromatography mass spectrometry (UPLC-MS; Acquity UPLC H-class with QDa detector; Waters) as described previously (33).

### Dialysate AA concentrations

Spent dialysate samples were collected in sterile tubes, immediately frozen in liquid nitrogen, and stored in a freezer at −80°C until further analysis. Collected dialysate was concentrated 5 times through freeze-drying 25 mL of the sample and dissolving the dried product in 5.0 mL 0.1 M hydrogen chloride. After homogenization, 50 µL of the concentrated sample was deproteinized using 100 µL of 10% SSA with 50 µM of the metabolomics amino acid mix MSK-A2 internal standard and processed in the same manner as plasma samples. Subsequently, AA profiles were determined through UPLC-MS.

### Calculations

AA loss in the dialysate (g) was calculated by multiplying the mean total amino acid (TAA) concentration of spent dialysate (grams per liter) with spent dialysate and ultrafiltration volume (liters). Essential amino acid (EAA) values are the sums of histidine, isoleucine, leucine, lysine, methionine, phenylalanine, threonine, tryptophan, and valine values. Nonessential amino acid (NEAA) values equal the sums of alanine, arginine, asparagine, aspartic acid, beta alanine, cystine, glutamic acid, glutamine, glycine, proline, serine, tryptophan, and tyrosine values. Branched-chain amino acid (BCAA) values are the totals of leucine, isoleucine, and valine values.

### Statistical analysis

All data are expressed as means ± SEM unless indicated otherwise. Time-dependent variables (i.e., TAA, EAA, and individual AA loss per 30 min) were analyzed by a 1-factor repeated-measures ANOVA. If a statistically significant time effect was found, post hoc paired-samples *t* tests were performed to locate the effects. Pre-HD and post-HD plasma AA concentrations were compared using paired-samples *t* tests. Correlations between dialysate AA concentrations and pre-HD plasma AA concentrations and dietary intake were assessed through determining the parametric Pearson's or the nonparametric Spearman's rank correlation coefficients for normally and nonormally distributed data, respectively. Statistical significance was set at *P* < 0.05. All analyses were performed using SPSS statistics software (version 24.0; IBM Corp.).

## Results

### Plasma AA concentrations

**FIGURE 1:**
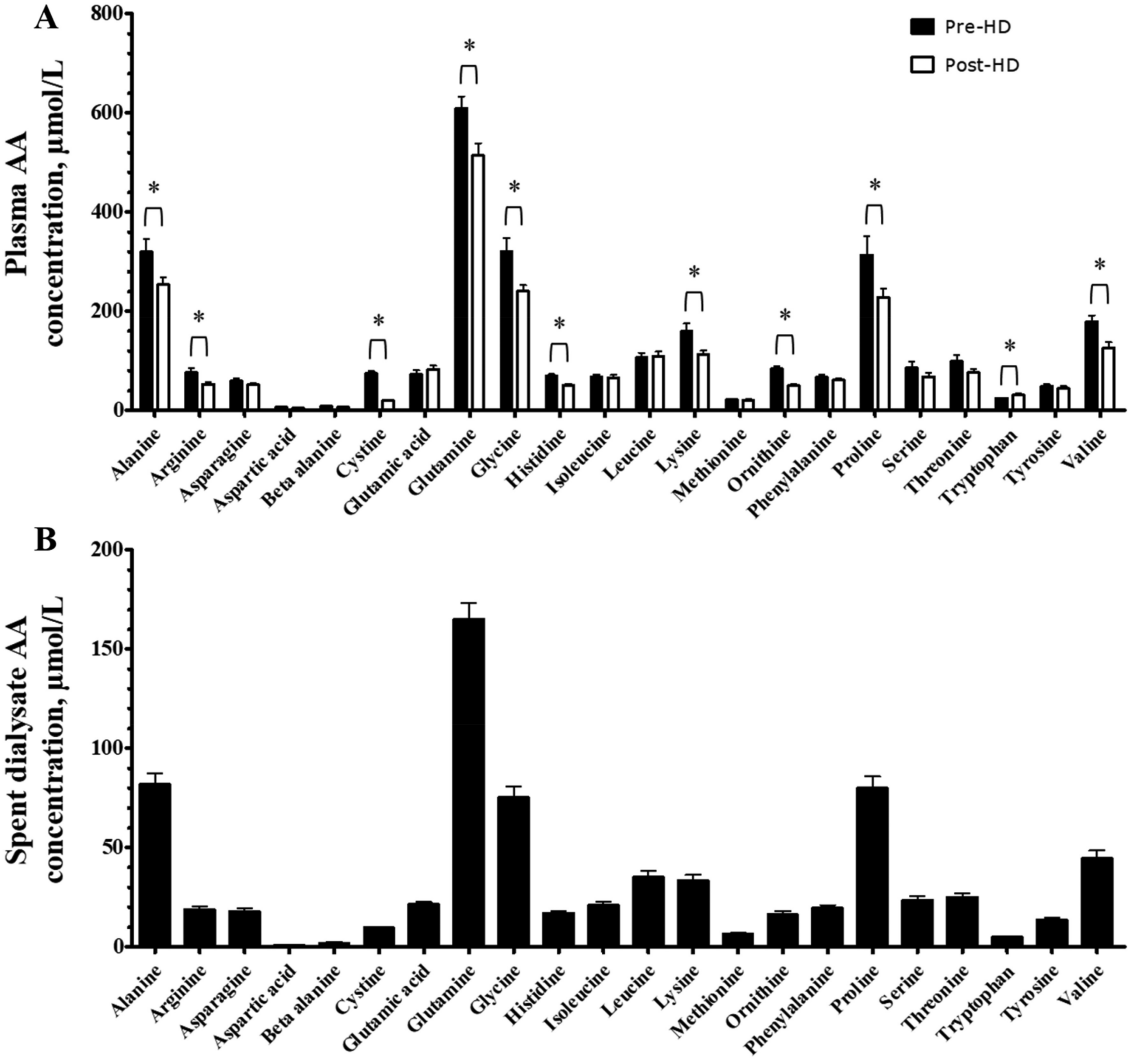
AA concentrations in (A) pre- and post-HD plasma and (B) spent dialysate of CHD patients. Plasma concentrations of 22 AAs are expressed as µmol/L. Values represent means + SEMs, *n* = 10. *Post-HD plasma AA concentrations are significantly different from pre-HD plasma AA concentrations, *P* < 0.05. AA, amino acid; CHD, chronic hemodialysis; HD, hemodialysis.

Pre-HD plasma TAA, NEAA, and EAA concentrations averaged 2.88 ± 0.15, 2.08 ± 0.11, and 0.80 ± 0.05 mmol/L, respectively. Post-HD plasma TAA, NEAA, and EAA concentrations were significantly reduced to 2.27 ± 0.11, 1.62 ± 0.07, and 0.66 ± 0.05 mmol/L, respectively (*P* < 0.05). Pre-HD and post-HD plasma BCAA concentrations were 0.35 ± 0.03 and 0.30 ± 0.03 mmol/L, respectively (*P* = 0.11). Whereas most individual AA concentrations decreased during HD, we observed a significant increase in plasma tryptophan concentrations (**Figure 1**A; *P* = 0.003).

### Spent dialysate AA concentrations

**FIGURE 2:**
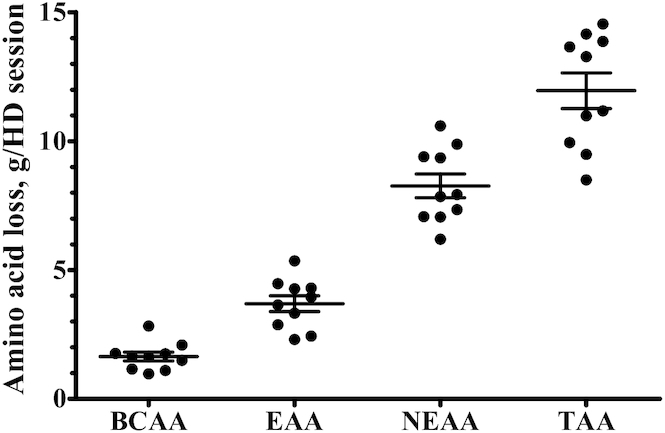
AA loss during a single HD session in CHD patients. Filled circles represent individual data points and bars represent group means ± SEMs, *n* = 10. AA, amino acid; BCAA, branched-chain amino acid; CHD, chronic hemodialysis; EAA, essential amino acid; HD, hemodialysis, NEAA, nonessential amino acid; TAA, total amino acid.

**FIGURE 3:**
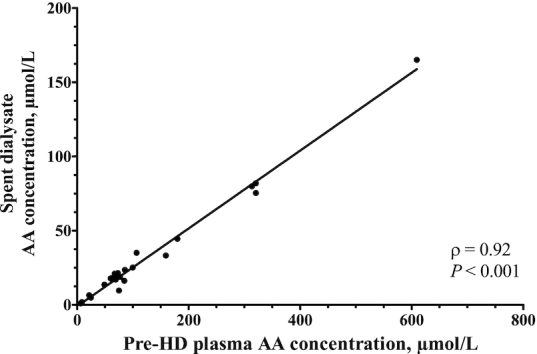
Correlation between AA profiles in spent dialysate and prehemodialysis plasma of CHD patients. AA concentrations are expressed as µmol/L, *n* = 10. Spearman's rank correlation coefficients were determined to assess correlations. AA, amino acid; CHD, chronic hemodialysis; HD, hemodialysis; TAA, total amino acid.

In the spent dialysate, the AAs with the highest and lowest average concentrations were glutamine and aspartic acid, respectively (Figure 1B). Spent dialysate TAA concentrations averaged 0.73 ± 0.03 mmol/L and did not differ between the 30-min sampling periods throughout the HD session (*P* = 0.94). Spent dialysate volume per HD session averaged 128 ± 5.05 L. TAA, NEAA, EAA, and BCAA losses during a single HD session are depicted in **Figure 2**. AA concentrations in spent dialysate were strongly correlated with pre-HD plasma AA concentrations (**Figure 3**; Spearman's ρ *=* 0.92, *P* < 0.001).

### Dietary intake prior to and during HD

**FIGURE 4:**
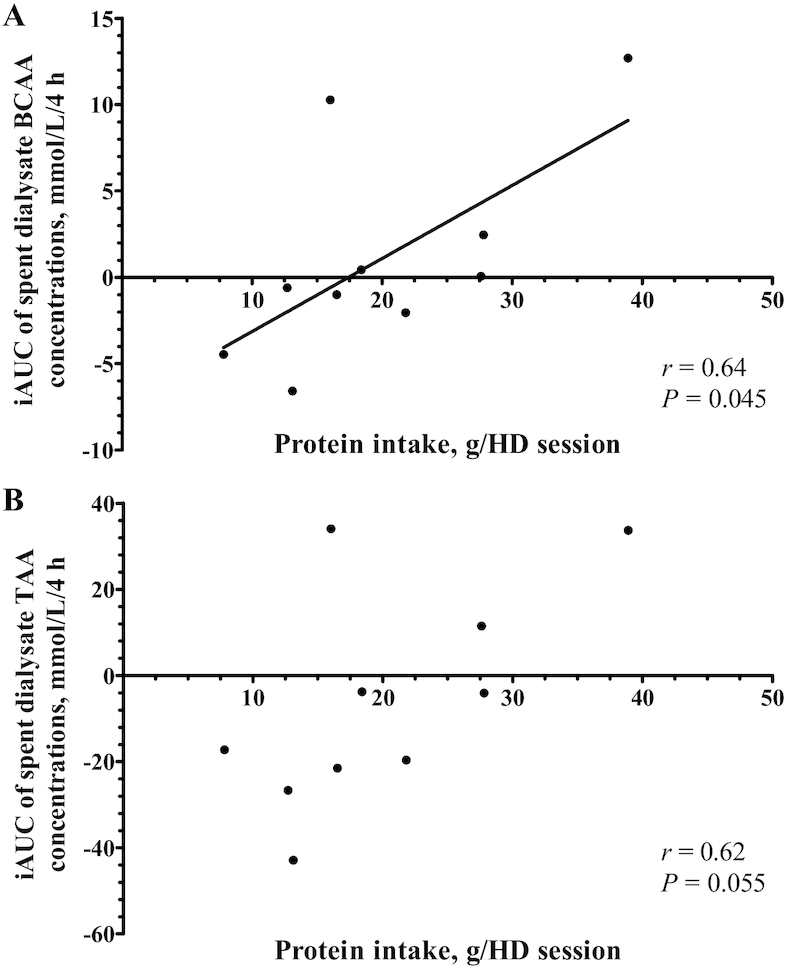
Correlations between protein intake during a single hemodialysis session and the iAUCs of (A) spent dialysate BCAA concentrations and (B) spent dialysate TAA concentrations in CHD. Protein intake levels are expressed as g/HD session, *n* = 10. Pearson's rank correlation coefficients were determined to assess correlations. AA, amino acid; CHD, chronic hemodialysis; BCAA, branched-chain amino acid; HD, hemodialysis; iAUC, incremental area under the curve; TAA, total amino acid.

Reported 24-h dietary protein and energy intakes averaged 1.03 ± 0.13 g/kg and 28.3 ± 2.9 kcal/kg, respectively (**Table 2**). All included patients consumed food and beverages during HD. Patients ingested 0.33 ± 0.05 g protein/kg and 8.9 ± 1.0 kcal/kg during a single HD session. Protein intake during HD was not associated with an attenuated decline in plasma AA concentrations over the HD session (*P* = 0.22). Protein intake was positively correlated with the incremental AUC of spent dialysate BCAA concentrations (**Figure 4**A; Pearson's *r =* 0.64, *P* = 0.045). Furthermore, the correlation of protein intake with the incremental AUC of spent dialysate TAA concentrations nearly reached statistical significance (Figure 4B; Pearson's *r =* 0.62, *P =* 0.055).

**TABLE 2:** Reported daily habitual energy and macronutrient intakes prior to and during HD in CHD patients consuming their habitual diet^1^

	24-h intake	During HD (4 h)
Energy, kcal	1786 ± 189	553 ± 53
Protein, g	64.6 ± 7.5	20.1 ± 2.9
Fat, g	72.6 ± 6.8	21.5 ± 3.1
Carbohydrates, g	213 ± 26.3	67.1 ± 6.5

1 All values are expressed as mean ± SEM, *n* = 10. CHD, chronic hemodialysis; HD, hemodialysis.

## Discussion

This is the first study to show that CHD patients ingesting their habitual diet throughout HD lose ∼12 g of AAs from the circulation during a single HD session. This is equivalent to the amount of AAs being released into the circulation following ingestion of a typical meal (containing 20–25g protein). The loss of AAs during HD results in a significant decline in circulating plasma AA concentrations.

HD is a life-saving treatment for end-stage renal disease patients with inadequate residual renal function (34). Besides harmful substances, HD also extracts small-sized nutrients from the circulation (11). We observed a decline in plasma concentrations of most AAs during HD, which resulted in an ∼20% decrease of plasma TAA concentrations. Individual changes in plasma TAA, NEAA, EAA, and BCAA concentrations throughout HD are depicted in **Supplemental Figure 1**. Plasma TAA concentrations after HD were ∼20% lower in our patients when compared with postabsorptive plasma TAA concentrations in healthy older adults observed in our laboratory recently (35). As depicted in Figure 1, AA profiles in pre-HD plasma and spent dialysate showed the same pattern. Accordingly, the correlation between AA concentrations in pre-HD plasma and spent dialysate was very strong (Figure 3). Thus, all AAs diffused through the HD membrane without selective restriction. During a single HD session, this resulted in an extraction of 11.95 ± 0.69 g AAs from the circulation, including ∼8 g NEAAs, ∼4 g EAAs, and ∼2 g BCAAs (Figure 2). This would be equivalent to the protein provided in a full meal containing 20–25 g protein, as only ∼50% of ingested dietary protein derived AAs typically reach the circulation during the first few hours after meal ingestion (22, 36).

It has been suggested that the extraction of AAs from the circulation may be compensated for through eating during HD (37). In the current study, patients ingested self-selected foods during HD *ad libitum*, as they would do during usual care. Despite a mean protein intake of 20 g throughout HD sessions, we observed a significant decline in plasma AA concentrations. An overview of individual food intake and spent dialysate TAA concentrations throughout HD is presented in **Supplemental Figure 2**. Protein ingestion has been shown to increase plasma AA concentrations in a dose-dependent manner (12, 16, 38), which most likely increases diffusion of AAs into the dialysate. In agreement, Veeneman et al. have previously shown that ingestion of protein-enriched meals throughout HD increases spent dialysate TAA concentrations (39). Increased AA extraction following food intake during HD may prevent patients consuming their habitual diet from maintaining their plasma AA concentrations throughout HD.

Current clinical guidelines recommend that patients undergoing HD consume at least 1.2 g protein/kg ideal body weight per day (40–42). However, most CHD patients fail to ingest this amount of protein (43, 44). In the current study, reported habitual dietary protein intake was ∼1.0 g/kg ideal body weight per day, and only 3 patients reported a protein intake of at least 1.2 g/kg ideal body weight per day. Inadequate dietary protein intake predisposes patients to the development and progression of protein malnutrition, which is frequently observed in CHD patients (45). Especially on dialysis days, dietary protein intake is important to compensate for the HD-induced extraction of AAs (46). However, throughout dialysis days habitual food ingestion patterns are typically disrupted due to time restrains and fatigue caused by the HD session (47, 48). These barriers to food intake result in a reduced dietary protein intake on dialysis days compared to nondialysis days (43). In many CHD patients, habitual dietary protein intake on dialysis days may not be sufficient to compensate for the HD-induced extraction of AAs, contributing to the depletion of body protein stores.

We would advocate that nutritional interventions to support muscle maintenance in CHD patients should aim to increase dietary protein intake on dialysis days. It has been suggested that protein intake on dialysis days can be increased through providing more protein-rich foods during HD (49). Furthermore, previous studies have shown that supplementation with 30–60 g protein can maintain plasma AA concentrations throughout HD (37–39, 50, 51). However, our results indicate that ingestion of a large protein dose during HD will also substantially increase AA extraction. Consequently, CHD patients who eat during HD should consume well over 1.2 g protein/kg (ideal) body weight on dialysis days to allow compensation for (additional) HD-based AA extraction. It remains to be established how much protein should be ingested during HD to optimally support muscle maintenance. To allow development of individualized nutritional guidelines for CHD patients, the impact of timing and distribution of protein ingestion throughout dialysis days still needs to be assessed.

In conclusion, 8–15 g of AAs are extracted from the circulation during a single HD session. In the current study, the habitual dietary protein intake of Dutch CHD patients during HD did not fully compensate for this loss, resulting in a significant decline in circulating plasma AA concentrations. This observed AA extraction contributes significantly to protein malnutrition in CHD patients and emphasizes the need to develop effective and individualized nutritional strategies to improve nutritional status in patients frequently undergoing HD.

## Supplementary Material

nxaa010_Supplemental_File

## Notes

Supported by a grant from the NUTRIM NWO Graduate Programme.

Author disclosures: The authors report no conflicts of interest.

Supplementary Figures 1 and 2 are available from the “Supplementary data” link in the online posting of the article and from the same link in the online table of contents at https://academic.oup.com/jn/.

Abbreviations used: AA, amino acid; BCAA, branched-chain amino acid; CHD, chronic hemodialysis; EAA, essential amino acid; HD, hemodialysis; iAUC, incremental area under the curve; NEAA, nonessential amino acid; SSA, 5-sulfosalicylic acid; TAA, total amino acid; UPLC-MS, ultra-performance liquid chromatography mass spectrometry.

## References

[1] Levey AS, Eckardt KU, Tsukamoto Y, Levin A, Coresh J, Rossert J, De Zeeuw D, Hostetter TH, Lameire N, Eknoyan G Definition and classification of chronic kidney disease: a position statement from Kidney Disease: Improving Global Outcomes (KDIGO). Kidney Int. 2005;67:2089–100. 15882252 10.1111/j.1523-1755.2005.00365.x

[2] Meyer TW, Hostetter TH Approaches to uremia. J Am Soc Nephrol. 2014;25:2151–8. 24812163 10.1681/ASN.2013121264 PMC4178448

[3] Obi Y, Qader H, Kovesdy CP, Kalantar-Zadeh K Latest consensus and update on protein-energy wasting in chronic kidney disease. Curr Opin Clin Nutr Metab Care. 2015;18:254–62. 25807354 10.1097/MCO.0000000000000171 PMC4506466

[4] Broers NJ, Usvyat LA, Kooman JP, van der Sande FM, Lacson E Jr., Kotanko P, Maddux FW Quality of life in dialysis patients: a retrospective cohort study. Nephron. 2015;130:105–12. 26044799 10.1159/000430814

[5] Marcelli D, Brand K, Ponce P, Milkowski A, Marelli C, Ok E, Merello Godino JI, Gurevich K, Jirka T, Rosenberger J et al. Longitudinal changes in body composition in patients after initiation of hemodialysis therapy: results from an international cohort. J Ren Nutr. 2016;26:72–80. 26627050 10.1053/j.jrn.2015.10.001

[6] Kooman JP, Kotanko P, Schols AM, Shiels PG, Stenvinkel P Chronic kidney disease and premature ageing. Nat Rev Nephrol. 2014;10:732–42. 25287433 10.1038/nrneph.2014.185

[7] Kooman JP, Broers NJ, Usvyat L, Thijssen S, van der Sande FM, Cornelis T, Levin NW, Leunissen KM, Kotanko P Out of control: accelerated aging in uremia. Nephrol Dial Transplant. 2013;28:48–54. 23139404 10.1093/ndt/gfs451

[8] Fouque D, Kalantar-Zadeh K, Kopple J, Cano N, Chauveau P, Cuppari L, Franch H, Guarnieri G, Ikizler TA, Kaysen G et al. A proposed nomenclature and diagnostic criteria for protein-energy wasting in acute and chronic kidney disease. Kidney Int. 2008;73:391–8. 18094682 10.1038/sj.ki.5002585

[9] Kopple JD. Pathophysiology of protein-energy wasting in chronic renal failure. J Nutr. 1999;129:247S–51S. 9915908 10.1093/jn/129.1.247S

[10] Lim VS, Bier DM, Flanigan MJ, Sum-Ping ST The effect of hemodialysis on protein metabolism. A leucine kinetic study. J Clin Invest. 1993;91:2429–36. 8514855 10.1172/JCI116477 PMC443302

[11] Alp Ikizler T, Flakoll PJ, Parker RA, Hakim RM Amino acid and albumin losses during hemodialysis. Kidney Int. 1994;46:830–7. 7996804 10.1038/ki.1994.339

[12] Pennings B, Groen B, de Lange A, Gijsen AP, Zorenc AH, Senden JM, van Loon LJ Amino acid absorption and subsequent muscle protein accretion following graded intakes of whey protein in elderly men. Am J Physiol Endocrinol Metab. 2012;302:E992–9. 22338070 10.1152/ajpendo.00517.2011

[13] Burd NA, Gorissen SH, van Vliet S, Snijders T, van Loon LJ Differences in postprandial protein handling after beef compared with milk ingestion during postexercise recovery: a randomized controlled trial. Am J Clin Nutr. 2015;102:828–36. 26354539 10.3945/ajcn.114.103184

[14] Kouw IW, Gorissen SH, Burd NA, Cermak NM, Gijsen AP, van Kranenburg J, van Loon LJ Postprandial protein handling is not impaired in type 2 diabetes patients when compared with normoglycemic controls. J Clin Endocrinol Metab. 2015;100:3103–11. 26037513 10.1210/jc.2015-1234

[15] Groen BB, Res PT, Pennings B, Hertle E, Senden JM, Saris WH, van Loon LJ Intragastric protein administration stimulates overnight muscle protein synthesis in elderly men. Am J Physiol Endocrinol Metab. 2012;302:E52–60. 21917635 10.1152/ajpendo.00321.2011

[16] Witard OC, Jackman SR, Breen L, Smith K, Selby A, Tipton KD Myofibrillar muscle protein synthesis rates subsequent to a meal in response to increasing doses of whey protein at rest and after resistance exercise. Am J Clin Nutr. 2014;99:86–95. 24257722 10.3945/ajcn.112.055517

[17] Mitchell WK, Phillips BE, Williams JP, Rankin D, Lund JN, Wilkinson DJ, Smith K, Atherton PJ The impact of delivery profile of essential amino acids upon skeletal muscle protein synthesis in older men: clinical efficacy of pulse vs. bolus supply. Am J Physiol Endocrinol Metab. 2015;309:E450–7. 26152764 10.1152/ajpendo.00112.2015

[18] Volpi E, Kobayashi H, Sheffield-Moore M, Mittendorfer B, Wolfe RR Essential amino acids are primarily responsible for the amino acid stimulation of muscle protein anabolism in healthy elderly adults. Am J Clin Nutr. 2003;78:250–8. 12885705 10.1093/ajcn/78.2.250 PMC3192452

[19] Drummond MJ, Bell JA, Fujita S, Dreyer HC, Glynn EL, Volpi E, Rasmussen BB Amino acids are necessary for the insulin-induced activation of mTOR/S6K1 signaling and protein synthesis in healthy and insulin resistant human skeletal muscle. Clin Nutr. 2008;27:447–56. 18342407 10.1016/j.clnu.2008.01.012 PMC2484120

[20] Luiking YC, Deutz NE, Memelink RG, Verlaan S, Wolfe RR Postprandial muscle protein synthesis is higher after a high whey protein, leucine-enriched supplement than after a dairy-like product in healthy older people: a randomized controlled trial. Nutr J. 2014;13:9. 24450500 10.1186/1475-2891-13-9 PMC3909458

[21] Bennet WM, Connacher AA, Scrimgeour CM, Smith K, Rennie MJ Increase in anterior tibialis muscle protein synthesis in healthy man during mixed amino acid infusion: studies of incorporation of [1-13C]leucine. Clin Sci (Lond). 1989;76:447–54. 2714054 10.1042/cs0760447

[22] Groen BB, Horstman AM, Hamer HM, de Haan M, van Kranenburg J, Bierau J, Poeze M, Wodzig WK, Rasmussen BB, van Loon LJ Post-prandial protein handling: you are what you just ate. PLoS One. 2015;10:e0141582. 26556791 10.1371/journal.pone.0141582 PMC4640549

[23] Raj DS, Dominic EA, Wolfe R, Shah VO, Bankhurst A, Zager PG, Ferrando A Coordinated increase in albumin, fibrinogen, and muscle protein synthesis during hemodialysis: role of cytokines. Am J Physiol Endocrinol Metab. 2004;286:E658–64. 14722024 10.1152/ajpendo.00444.2003

[24] Ikizler TA, Pupim LB, Brouillette JR, Levenhagen DK, Farmer K, Hakim RM, Flakoll PJ Hemodialysis stimulates muscle and whole body protein loss and alters substrate oxidation. Am J Physiol Endocrinol Metab. 2002;282:E107–16. 11739090 10.1152/ajpendo.2002.282.1.E107

[25] Navarro JF, Mareen R, Teruel JL, del Rio RM, Gamez C, Mora C, Ortuno J Effect of different membranes on amino-acid losses during haemodialysis. Nephrol Dial Transplant. 1998;13:113–7. 9481725 10.1093/ndt/13.1.113

[26] Wolfson M, Jones MR, Kopple JD Amino acid losses during hemodialysis with infusion of amino acids and glucose. Kidney Int. 1982;21:500–6. 7087285 10.1038/ki.1982.52

[27] Gil HW, Yang JO, Lee EY, Lee EM, Choi JS, Hong SY The effect of dialysis membrane flux on amino acid loss in hemodialysis patients. J Korean Med Sci. 2007;22:598–603. 17728495 10.3346/jkms.2007.22.4.598 PMC2693805

[28] Yokomatsu A, Fujikawa T, Toya Y, Shino-Kakimoto M, Itoh Y, Mitsuhashi H, Tamura K, Hirawa N, Yasuda G, Umemura S Loss of amino acids into dialysate during hemodialysis using hydrophilic and nonhydrophilic polyester-polymer alloy and polyacrylonitrile membrane dialyzers. Ther Apher Dial. 2014;18:340–6. 24206420 10.1111/1744-9987.12145

[29] Chazot C, Shahmir E, Matias B, Laidlaw S, Kopple JD Dialytic nutrition: provision of amino acids in dialysate during hemodialysis. Kidney Int. 1997;52:1663–70. 9407515 10.1038/ki.1997.500

[30] Kistler B, Benner D, Burgess M, Stasios M, Kalantar-Zadeh K, Wilund KR To eat or not to eat—international experiences with eating during hemodialysis treatment. J Ren Nutr. 2014;24:349–52. 25443543 10.1053/j.jrn.2014.08.003

[31] Broers NJH, Martens RJH, Cornelis T, van der Sande FM, Diederen NMP, Hermans MMH, Wirtz J, Stifft F, Konings C, Dejagere T et al. Physical activity in end-stage renal disease patients: the effects of starting dialysis in the first 6 months after the transition period. Nephron. 2017;137:47–56. 28591752 10.1159/000476072 PMC5872558

[32] National Institute of Public Health MoH, Welfare and Sport. Dutch Food Composition Database. 2016; [cited 2018 May 5]. Available from: https://nevo-online.rivm.nl.

[33] Waterval WA, Scheijen JL, Ortmans-Ploemen MM, Habets-van der Poel CD, Bierau J Quantitative UPLC-MS/MS analysis of underivatised amino acids in body fluids is a reliable tool for the diagnosis and follow-up of patients with inborn errors of metabolism. Clin Chim Acta. 2009;407:36–42. 19559691 10.1016/j.cca.2009.06.023

[34] Himmelfarb J, Ikizler TA Hemodialysis. N Engl J Med. 2010;363:1833–45. 21047227 10.1056/NEJMra0902710

[35] Fuchs CJ, Hermans WJH, Holwerda AM, Smeets JSJ, Senden JM, van Kranenburg J, Gijsen AP, Wodzig W, Schierbeek H, Verdijk LB et al. Branched-chain amino acid and branched-chain ketoacid ingestion increases muscle protein synthesis rates in vivo in older adults: a double-blind, randomized trial. Am J Clin Nutr. 2019;110:862–72. 31250889 10.1093/ajcn/nqz120 PMC6766442

[36] van Vliet S, Skinner SK, Beals JW, Pagni BA, Fang HY, Ulanov AV, Li Z, Paluska SA, Mazzulla M, West DWD et al. Dysregulated handling of dietary protein and muscle protein synthesis after mixed-meal ingestion in maintenance hemodialysis patients. Kidney Int Rep. 2018;3:1403–15. 30450467 10.1016/j.ekir.2018.08.001 PMC6224635

[37] Kistler BM, Benner D, Burrowes JD, Campbell KL, Fouque D, Garibotto G, Kopple JD, Kovesdy CP, Rhee CM, Steiber A et al. Eating during hemodialysis treatment: a consensus statement from the International Society of Renal Nutrition and Metabolism. J Ren Nutr. 2018;28:4–12. 29249295 10.1053/j.jrn.2017.10.003

[38] Sundell MB, Cavanaugh KL, Wu P, Shintani A, Hakim RM, Ikizler TA Oral protein supplementation alone improves anabolism in a dose-dependent manner in chronic hemodialysis patients. J Ren Nutr. 2009;19:412–21. 19500999 10.1053/j.jrn.2009.01.019 PMC2758490

[39] Veeneman JM, Kingma HA, Boer TS, Stellaard F, De Jong PE, Reijngoud DJ, Huisman RM Protein intake during hemodialysis maintains a positive whole body protein balance in chronic hemodialysis patients. Am J Physiol Endocrinol Metab. 2003;284:E954–65. 12540372 10.1152/ajpendo.00264.2002

[40] Kopple JD. National Kidney Foundation K/DOQI clinical practice guidelines for nutrition in chronic renal failure. Am J Kidney Dis. 2001;37:S66–70. 11158865 10.1053/ajkd.2001.20748

[41] Ikizler TA, Cano NJ, Franch H, Fouque D, Himmelfarb J, Kalantar-Zadeh K, Kuhlmann MK, Stenvinkel P, TerWee P, Teta D et al. Prevention and treatment of protein energy wasting in chronic kidney disease patients: a consensus statement by the International Society of Renal Nutrition and Metabolism. Kidney Int. 2013;84:1096–107. 23698226 10.1038/ki.2013.147

[42] Deutz NE, Bauer JM, Barazzoni R, Biolo G, Boirie Y, Bosy-Westphal A, Cederholm T, Cruz-Jentoft A, Krznaric Z, Nair KS et al. Protein intake and exercise for optimal muscle function with aging: recommendations from the ESPEN Expert Group. Clin Nutr. 2014;33:929–36. 24814383 10.1016/j.clnu.2014.04.007 PMC4208946

[43] Martins AM, Dias Rodrigues JC, de Oliveira Santin FG, Barbosa Brito Fdos S, Bello Moreira AS, Lourenco RA, Avesani CM Food intake assessment of elderly patients on hemodialysis. J Ren Nutr. 2015;25:321–6. 25572139 10.1053/j.jrn.2014.10.007

[44] Bossola M, Tazza L, Giungi S, Luciani G Anorexia in hemodialysis patients: an update. Kidney Int. 2006;70:417–22. 16775598 10.1038/sj.ki.5001572

[45] Carrero JJ, Thomas F, Nagy K, Arogundade F, Avesani CM, Chan M, Chmielewski M, Cordeiro AC, Espinosa-Cuevas A, Fiaccadori E et al. Global prevalence of protein-energy wasting in kidney disease: a meta-analysis of contemporary observational studies from the international society of renal nutrition and metabolism. J Ren Nutr. 2018;28:380–92. 30348259 10.1053/j.jrn.2018.08.006

[46] Borah MF, Schoenfeld PY, Gotch FA, Sargent JA, Wolfson M, Humphreys MH Nitrogen balance during intermittent dialysis therapy of uremia. Kidney Int. 1978;14:491–500. 750694 10.1038/ki.1978.154

[47] Clark-Cutaia MN, Sevick MA, Thurheimer-Cacciotti J, Hoffman LA, Snetselaar L, Burke LE, Zickmund SL Perceived barriers to adherence to hemodialysis dietary recommendations. Clin Nurs Res. 2019;28(8):1009–29. 29732932 10.1177/1054773818773364

[48] St-Jules DE, Woolf K, Pompeii ML, Sevick MA Exploring problems in following the hemodialysis diet and their relation to energy and nutrient intakes: The Balance Wise Study. J Ren Nutr. 2016;26:118–24. 26586249 10.1053/j.jrn.2015.10.002 PMC4762735

[49] Struijk-Wielinga GI, Romijn M, Neelemaat F, ter Wee PM, Weijs PJM Providing in-between meals during dialysis treatment contributes to an adequate protein and energy intake in hemodialysis patients: a non-randomized intervention study. M J Nutr. 2016;1(1):006.

[50] Pupim LB, Majchrzak KM, Flakoll PJ, Ikizler TA Intradialytic oral nutrition improves protein homeostasis in chronic hemodialysis patients with deranged nutritional status. J Am Soc Nephrol. 2006;17:3149–57. 17021267 10.1681/ASN.2006040413

[51] Martin-Alemany G, Valdez-Ortiz R, Olvera-Soto G, Gomez-Guerrero I, Aguire-Esquivel G, Cantu-Quintanilla G, Lopez-Alvarenga JC, Miranda-Alatriste P, Espinosa-Cuevas A The effects of resistance exercise and oral nutritional supplementation during hemodialysis on indicators of nutritional status and quality of life. Nephrol Dial Transplant. 2016;31:1712–20. 27510532 10.1093/ndt/gfw297

