# Supplementary material for: End-Stage Renal Disease Patients Lose a Substantial Amount of Amino Acids during Hemodialysis
Source: J Nutr. 2020 Jan 31;150(5):1160–6. doi: 10.1093/jn/nxaa010 (PMC7198312; doi:10.1093/jn/nxaa010)
Supplement: nxaa010_Supplemental_File [file nxaa010_supplemental_file.docx]

**End-Stage Renal Disease Patients Lose a Substantial Amount of Amino Acids during
Hemodialysis**

Floris K. Hendriks, Joey S.J. Smeets, Natascha J.H. Broers, Janneau M.X. van Kranenburg, Frank M. van der Sande, Jeroen P. Kooman, and Luc J.C. van Loon.

**Online Supplementary Material**

**SUPPLEMENTAL FIGURE 1**

**
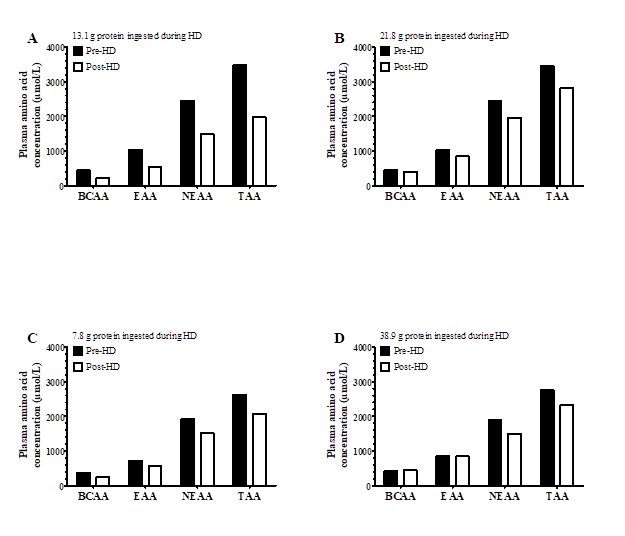
**

**SUPPLEMENTAL FIGURE 1 (continued)**
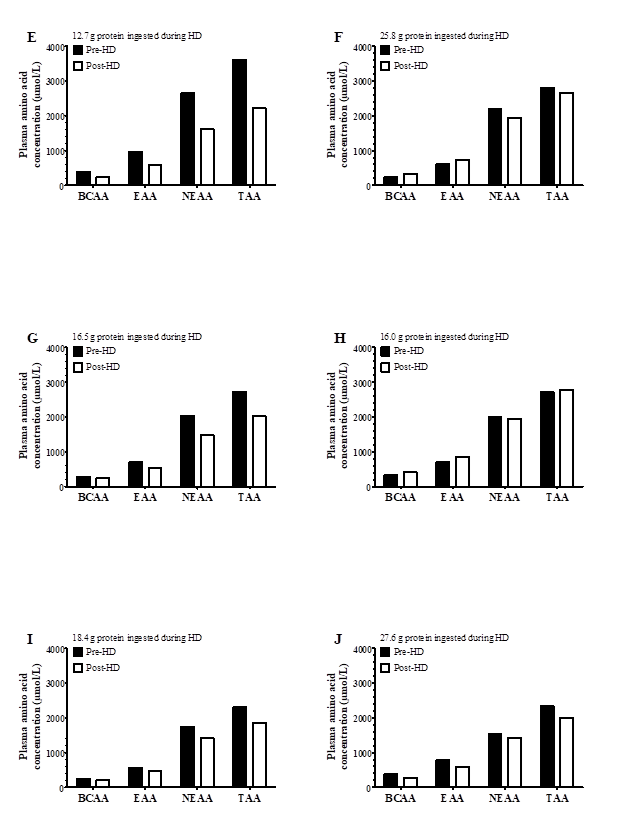


**Individual pre-HD and post-HD plasma total amino acid concentrations and protein intake throughout a hemodialysis session in ten chronic hemodialysis patients.** Protein intake was achieved through habitual dietary consumption and is expressed as g, and plasma total amino acid concentrations are expressed as µmol/L, *n*=10. BCAA, branched-chain amino acid; EAA, essential amino acid; HD, hemodialysis, NEAA, non-essential amino acid; TAA, total amino acid.

**SUPPLEMENTAL FIGURE 2**

**SUPPLEMENTAL FIGURE 2 (continued)** ****

**Individual spent dialysate amino acid concentrations and protein intake throughout a hemodialysis session in ten chronic hemodialysis patients.** Concentrations in spent dialysate are expressed as µmol/L and represent the mean concentration of the 30 min prior to sampling. Protein and energy intake were achieved through habitual dietary consumption and are expressed as g and kcal, respectively. *t*= 0 min represents the start of the hemodialysis session. TAA, total amino acid.
